# The relationship between maternal education and mortality among women giving birth in health care institutions: Analysis of the cross sectional WHO Global Survey on Maternal and Perinatal Health

**DOI:** 10.1186/1471-2458-11-606

**Published:** 2011-07-29

**Authors:** Saffron Karlsen, Lale Say, João-Paulo Souza, Carol J Hogue, Dinorah L Calles, A Metin Gülmezoglu, Rosalind Raine

**Affiliations:** 1 Department of Epidemiology & Public Health, UCL, 1-19 Torrington Place, London WC1E 6BT, UK; 2 Department of Reproductive Health and Research, World Health Organisation, Avenue Appia 20, Geneva, CH-1211, Switzerland; 3 Rollins School of Public Health, Emory University, 1518 Clifton Rd NE, Atlanta, GA 30322, USA

## Abstract

**Background:**

Approximately one-third of a million women die each year from pregnancy-related conditions. Three-quarters of these deaths are considered avoidable. Millennium Development Goal five calls for a reduction in maternal mortality and the establishment of universal access to high quality reproductive health care. There is evidence of a relationship between lower levels of maternal education and higher maternal mortality. This study examines the relationship between maternal education and maternal mortality among women giving birth in health care institutions and investigates the association of maternal age, marital status, parity, institutional capacity and state-level investment in health care with these relationships.

**Methods:**

Cross-sectional information was collected on 287,035 inpatients giving birth in 373 health care institutions in 24 countries in Africa, Asia and Latin America, between 2004-2005 (in Africa and Latin America) and 2007-2008 (in Asia) as part of the WHO Global Survey on Maternal and Perinatal Health. Analyses investigated associations between indicators measured at the individual, institutional and country level and maternal mortality during the intrapartum period: from admission to, until discharge from, the institution where women gave birth. There were 363 maternal deaths.

**Results:**

In the adjusted models, women with no education had 2.7 times and those with between one and six years of education had twice the risk of maternal mortality of women with more than 12 years of education. Institutional capacity was not associated with maternal mortality in the adjusted model. Those not married or cohabiting had almost twice the risk of death of those who were. There was a significantly higher risk of death among those aged over 35 (compared with those aged between 20 and 25 years), those with higher numbers of previous births and lower levels of state investment in health care. There were also additional effects relating to country of residence which were not explained in the model.

**Conclusions:**

Lower levels of maternal education were associated with higher maternal mortality even amongst women able to access facilities providing intrapartum care. More attention should be given to the wider social determinants of health when devising strategies to reduce maternal mortality and to achieve the increasingly elusive MDG for maternal mortality.

## Background

It is estimated that each year approximately one third of a million women worldwide die due to pregnancy-related conditions [1]. 99% of these deaths occur in developing countries and approximately three-quarters of them are considered avoidable [2]. Millennium Development Goal five (MDG5) calls for a reduction in the maternal mortality ratio (i.e. the number of maternal deaths per 100,000 live births) by three quarters by 2015 and the establishment of universal access to high quality reproductive health care [3-5]. Achievement of MDG5 in part requires improved provision of family planning services to enable women to have fewer, better spaced pregnancies [6]. However major causes of maternal death include intra-partum emergencies such as haemorrhage, obstructed labour and infections [7], and these also need to be addressed. Only a third of women who require lifesaving care following a complication in delivery receive it [8]. It is therefore argued that a vital prerequisite to reducing maternal deaths is universal access to high quality pregnancy and delivery care [9]. This includes an appropriate and effective referral system and emergency obstetric care, including blood, medications and obstetric surgeries close to the people [3].

The consensus view is that a series of well tested interventions and a continuum of care should be the principal focus for efforts to reduce maternal mortality [6,9]. However, findings from the recent WHO Commission on Social Determinants of Health draw renewed attention to the need for the link between women's socio-economic characteristics and health to also be considered [10]. The more socially and economically advantaged people are, the better their health. Years of formal education are a well-recognised indicator of social position and have been frequently used in international surveys to explore social inequalities [11]. These studies show that people with progressively more advanced levels of education have better health and longer lives than those without [11]. But it has been argued that women's education should not be treated merely as a proxy for the social determinants of health but as an important force in its own right [12]. Women's educational levels (relative to those of men) have been found to be associated with maternal death [13]. There is a positive relationship between levels of maternal education and health service use,[14] even in adverse family or socioeconomic situations [15]. Furthermore, lack of education is highlighted as one of a number of stressors (along with limited money and decision-making power) affecting women during pregnancy and childbirth, creating vulnerability and increasing the likelihood of negative outcomes [16]. It is possible that much of the health disadvantage associated with low levels of maternal education can be addressed through universal access to quality health services; however, this hypothesis has not been tested empirically.

It is now recognised that MDG5 is highly unlikely to be achieved by 2015 [17]. It is maintained that a key stumbling block is the inability to establish and maintain robust health systems with appropriate obstetric facilities where they are most needed [3]. In view of the recognition of the role of education as a factor in mortality decline, we examined the contribution of maternal education to maternal mortality amongst women who were able to deliver in health care facilities and whether this contribution is attenuated by the services available in those institutions. We take advantage of the unique opportunities in the international data from the World Health Organisation (WHO) Global Survey on Maternal and Perinatal Health to investigate the relationship between the level of health care services available in institutions where women gave birth, women's educational level and maternal mortality. Our hypothesis was that the statistical relationship between maternal education and mortality would not be attenuated by adjusting for the effects of the services available in the institutions where women give birth.

## Methods

The WHO Global Survey on Maternal and Perinatal Health is a multi-country, health care facility-based, cross-sectional survey that collected data for all women giving birth in 373 randomly selected facilities from 24 randomly selected countries in Africa, Latin America and Asia. The methodological details of the survey have been published elsewhere [18,19]. In brief, the primary research was conducted using a stratified multistage cluster sampling design in order to obtain a sample of countries and health institutions worldwide. Countries in the WHO regions were further grouped according to their mortality levels for adults and those under five years of age. From each of these sub-regions, countries were selected, with probability proportional to population size. In each selected country, two regions or provinces, in addition to the capital city, were randomly selected with probability of selection proportional to their population size. In each selected province, a census of all health care facilities with more than 1000 births per year and those able to perform caesarean sections was obtained and seven facilities were randomly selected by computer, with probability of selection proportional to the number of births per year. Written permission from all Ministries of Health of the participating countries and the Directors of the selected facilities was obtained. The WHO Ethics Review Committee and that of each country independently approved the protocol.

Data collection took place between 2004-2005 in countries in Africa and Latin America and between 2007-2008 in Asia, over a period of two or three months depending on institutional delivery numbers. All women having a delivery after more than 20 weeks gestation during the data collection period in the selected facilities were included in the study. Women who died before delivery or who died having been referred postpartum to the health facility were excluded. All maternal deaths taking place after hospital discharge or after the seventh postpartum day were not captured.

Information on the demographic and health characteristics, pregnancy, delivery and maternal and perinatal outcomes (up to discharge) of individual women was obtained from medical records. Trained data clerks reviewed the medical records of all women giving birth at the institution and of their babies during the study period. Data describing each facility were collected on a standard proforma by the hospital coordinator in consultation with the Director or Head of Obstetrics. Random checks comparing collected data and hospital records and internal consistency checks were performed. Institutional data collected included the availability of basic services (e.g. reliable water supply, sterilization equipment, a blood bank), general medical services (including adult and neonatal intensive care), laboratory facilities for analysing antenatal screening tests; anaesthesiology resources; emergency obstetric services, intrapartum facilities (including the availability of staff skilled in caesarean section and ultrasound), the level of clinically trained staff and the presence of clinical protocols.

### Measures

Our main outcome was maternal mortality during the intrapartum period. Mortality data was available for 286,620 individuals.

Our main independent variable was the number of years of formal education received by the mother, categorised according to the UNESCO international standard classification of education [20]. This classification allocates individuals to one of five groups which correspond to the level of education expected after a given number of years of education: no education (zero years of education); primary (between one and six years of education); lower secondary (between seven and nine years of education); upper secondary (between 10 and 12 years of education); post-secondary/tertiary (more than 12 years of education). Information on education was available for 272,138 individuals.

We included a summary index of Institutional Capacity, which was used in the original study to determine the level of services available in each of the facilities to summarise an institution's capacity to provide obstetric care [21]. This Index comprised eight categories reflecting the: standard of building/basic services, maternal intrapartum care and human resources; availability of general medical care, anaesthesiology, emergency obstetric services; and provision of screening tests and academic resources and clinical protocols. The Index is determined by allocating two points to an institution for each of twenty eight 'Essential' services and one for each of nine 'Additional' services available (Table 1). We then calculated an overall unweighted score for each institution. Only those with no missing data on any item were included in the index. There was a potential maximum score of 56 points for institutions where every 'Essential' service was available and 66 points for those institutions where every 'Essential' and every 'Additional' service was available. Scores for the sampled institutions ranged from eight to 63 points. The scores were included as a continuous variable. Index scores were available for 269,260 women.

**Table 1:** Indicators included in the Institutional Capacity Index

Category	Essential	Additional
Basic Services	Availability of:	Availability of:
	• Reliable water supply	• Telephone
	• Sanitation facilities	
	• Electricity	
	• Generator	
	• Refrigerator	
	• Sterilization equipments	
General Medical Services	• Blood bank	• Medical clinics for referral in the same building
	• Safe blood	
	• Biochemical/Clinical laboratories	
	• High risk pregnancy beds	
	• Adult intensive care unit	
	• Neonatal intensive care unit	
Screening Tests	• Proteinuria	• Pap smear
	• HIV	• Hepatitis B
	• Syphilis	• Glucose tolerance test
	• Urine Culture	
Anaesthesiology Resources	• Anaesthesiologist or nurse/paramedics anaesthetist in hospital or on call	• Anaesthesiologist 24 hours per day in hospital
Emergency Obstetric Services	• Parenteral antibiotics	
	• Parenteral oxytocic drugs	
	• Parenteral anticonvulsants for pre-eclampsia and eclampsia	
	• Blood transfusion	
	• Neonatal resuscitation	
	• Maternal cardio pulmonary resuscitation	
	• Hysterectomy	
Intrapartum Care	• Partograph	• Fetal/obstetric ultrasound
	• Staff skilled in caesarean section	
Human Resources	• At least one obstetrics/gynaecology specialist	• At least one resident MD in training
Academic Resources and Clinical Protocols	• Formal protocols for Intrapartum care	• Continuous medical education programme

Other independent variables included in the analysis were maternal age (N = 286,360), marital status (coded married or not (N = 286,023), number of previous births (N = 286,087) and indicators of national health system. The effects of maternal age were investigated using continuous (quadratic) and categorical approaches (coded as age 10-19, 20-25, 26-30, 31-35 and 36 years and over). Maternal age, marital status, number of previous births and national health system have all been previously shown to be associated with maternal mortality [9,22,23]. Preliminary analyses suggested that the relationships between maternal mortality and maternal age were not linear. A grouped age variable was employed in the fully-adjusted models to allow greater insight into the nature of these relationships, and for clarity of interpretation.

We took account of the national health system in each country, using a typology based on the national economic level and the extent of state responsibility for the provision and organisation of health services [24]. Following this typology, we identified two indicators at the country level: 'level of health resources' (defined as the per capita health expenditure in international dollars in 2006) and 'level of state responsibility for health' (defined as public expenditure as percent of total health expenditure in 2006). Both indicators were included as continuous variables. This information was available for all countries included in the sample.

Preliminary regression analyses showed collinearity between the indicators exploring national public expenditure on health care and national per capita health expenditure, suggesting considerable overlap between the two measures in terms of their relationship with maternal mortality. Findings showed that the indicator of state responsibility for health had a greater influence on maternal mortality than that for level of health resources. Only the indicator for level of state responsibility was included in the model.

Continuous indicators of maternal age (used only in bivariate models), number of previous births, institutional capacity and level of state responsibility for health were each centred around their mean. This statistical adjustment enhances the interpretation of the analyses because it focuses the findings on variations from the sample means. Individuals with item-specific missing were included in the analyses.

### Analysis

We employed a series of logistic regression analyses to investigate univariate relationships between maternal mortality and each of the independent variables. Those with more than 12 years of education and those aged between 20 and 25 years old were reference categories for their respective analyses.

We then applied a multilevel model to explore the combined impact of each of the independent variables on maternal mortality. This model comprised three levels - individual mother, health care facility and country - to allow for the clustered nature of the sample. This random effects model generalises the ordinary fixed-effects logistic (regression) model by assuming that the individual probabilities of maternal mortality are equal to the fixed-effects model plus random variation due to unobserved, or unmeasured, effects relating to the institution and country.

We applied a three-level random effects logistic regression model to investigate the factors influencing maternal mortality. We estimate *p_ijk _*, the probability of maternal mortality for the ith respondent in the jth facility in the kth country, using a vector of covariates corresponding to the ith respondent in the jth facility in the kth country. The fixed effects included at the individual level of the multilevel model are maternal education, marital status, parity, maternal age. Public expenditure on health care was included as a fixed effect at the country level. The Institutional Capacity Index was included at the health-care-facility level of the multilevel model, with a random slope varying with country. The models employed here allowed for national variations in basic levels of maternal mortality (using a random country-specific intercept) and in the relationship between the Institutional Capacity Index and maternal mortality (using a random country-specific slope for the Index). The random effects were given a multivariate normal distribution, with mean zero and covariance matrix which accounts for correlation between slope and intercept. When σ_u _= 0, the model reduces to the ordinary logistic model, indicating that there was no significant correlation in the risk of maternal mortality between country. The significance of the random effects terms was tested with a modified likelihood ratio test by comparing the null hypothesis σ_u _= 0 against the alternative hypothesis σ_u _> 0 ([25,26]). Logistic regression analyses were conducted using Stata version 9. Multilevel modelling was conducted using MLwin version 2.12.

## Results

We analysed data on 287,035 women, of whom 363 had died before being discharged from the institution where they delivered (Table 2). One country (Japan) had no maternal deaths during the period. Of those countries with maternal deaths, 14 had fewer than ten, two had between ten and 20, four had between 20 and 40, and three had between 40 and 75. The deaths were distributed across the three regions, though not evenly: 25 occurred in Latin America, 100 in Asia and 238 in Africa. Intrapartum maternal mortality rates varied between the regions. Rates in Africa (290 per 100,000 women) were more than three times as high as in Asia (at 90 per 100,000 women) and almost ten times as high as in Latin America (at 30 per 100,000 women) (Table 1; Additional File 1, Table S1).

**Table 2:** Characteristics of the women, institutions and countries sampled, classified by region

	Africa (N = 81,958)	Latin America (N = 97,095)	Asia (N = 107,982)	Total (N = 287,035)
	% (n)	% (n)	% (n)	% (n)
**Characteristics of the women**				
Maternal death	0.29 (238)	0.03 (25)	0.09 (100)	0.13 (363)
				
UNESCO International Standard Classification of Education^21^:				
No education	14.79 (11002)	1.61 (1486)	8.42 (8893)	7.86 (21381)
Primary education (1-6 years of education)	19.96 (14851)	24.46 (22536)	18.02 (19033)	20.73 (56420)
Lower secondary (7-9 years of education)	26.51 (19721)	25.71 (23681)	21.57 (22783)	24.32 (66185)
Upper secondary (10-12 years of education)	25.18 (18734)	36.91 (34002)	35.18 (37155)	33.03 (89891)
Post-secondary/tertiary (>12 years of education)	13.56 (10090)	11.31 (10420)	16.81 (17751)	14.06 (38261)
Mean(SE) number of years of education	7.99 (0.02)	9.23 (0.01)	9.04 (0.01)	8.81 (0.01)
Range: min, 25%, 75%, max	0, 6, 12, 28	0, 6, 12, 28	0, 6, 12, 29	0, 6, 12, 29
				
Maternal age				
10-19 years	14.12 (11486)	18.26 (17718)	6.18 (6672)	12.53 (35876)
20-25 years	33.84 (27526)	34.56 (33543)	42.15 (45507)	37.22 (106576)
26-30 years	25.89 (21059)	23.21 (22522)	30.26 (32675)	26.63 (76256)
31-35 years	16.07 (13076)	15.52 (15066)	14.71 (15878)	15.37 (44020)
36-55 years	10.09 (8205)	8.56 (8196)	6.70 (7231)	8.25 (23632)
Mean(SE) age	26.60 (0.02)	25.76 (0.02)	26.51 (0.02)	26.28 (0.01)
				
Marital status:				
Single	11.70 (9517)	21.51 (20807)	5.71 (6159)	12.76 (36483)
Separated/divorced	0.58 (473)	0.83 (807)	0.51 (549)	0.64 (1829)
Married/cohabiting	87.47 (71153)	77.18 (74654)	93.65 (101095)	86.32 (246902)
Widowed/other	0.25 (203)	0.47 (455)	0.14 (151)	0.28 (809)
				
Mean(SE) number of previous births	1.76 (0.01)	1.12 (0.00)	0.76 (0.00)	1.16 (0.00)
				
**Characteristics of the institutions**				
Mean(SE) score on facility capacity index	49.15 (0.04)	58.85 (0.02)	57.85 (0.02)	55.84 (0.02)
Range: min, 25%, 75%, max	8, 43, 59, 63	21, 57, 63, 63	32, 54, 62, 63	8, 53, 61, 63
				
**Characteristics of the countries**				
Mean(SE) national per capita health expenditure ($, 2006)	109.59 (0.36)	619.67 (0.90)	217.71 (1.29)	322.81 (0.71)
Range: min, 25%, 75%, max	19, 38, 71, 315	235, 316, 674, 1205	52, 86, 171, 2581	19, 71, 316, 2581
				
Mean(SE) national public expenditure (as % of total health expenditure, 2006)	48.94 (0.08)	53.37 (0.05)	37.89 (0.04)	46.28 (0.04)
Range: min, 25%, 75%, max	19, 25, 81, 87	38, 44, 58, 92	25, 26, 48, 81	19, 32, 55, 92

SE = Standard Error.

There were differences in the number of years of formal education received by women in the three regions (Table 2). Almost 15% of the women attending health care facilities in Africa received no formal education compared with 8.4% of those in Asia and 1.6% of those in Latin America. However, the inter-quartile range of total years of education was similar across the three regions. Women attending health care facilities in Latin America had a slightly lower mean age than those in Africa and Asia. Women attending health care facilities in Asia were most likely to be married or cohabiting, and those in Latin America were most likely to be single. In Latin America, 22% of women attending health care facilities were single, compared with 12% of women in Africa and 6% in Asia. Women attending health care facilities in Asia had the lowest number of previous births and women attending health care facilities in Africa had the highest number.

The health care facilities sampled in Africa had a lower mean Capacity Index score than those in Asia and Latin America (Table 2). Almost 75% of institutions in the African countries sampled and more than 25% of those in Latin America and Asia lacked the full range of 'Essential' services for intrapartum care (and did not achieve the maximum score of 56 points available for institutions with every 'Essential' service).

The mean national per capita health expenditure in the Latin American countries examined was five and a half times that in the African countries examined (Table 2). The expenditure in the Asian countries examined was twice that in the African countries examined. The mean national public expenditure on health care, as a proportion of total health expenditure, was lowest in the Asian countries and highest in the Latin American countries examined.

**Figure 1:**
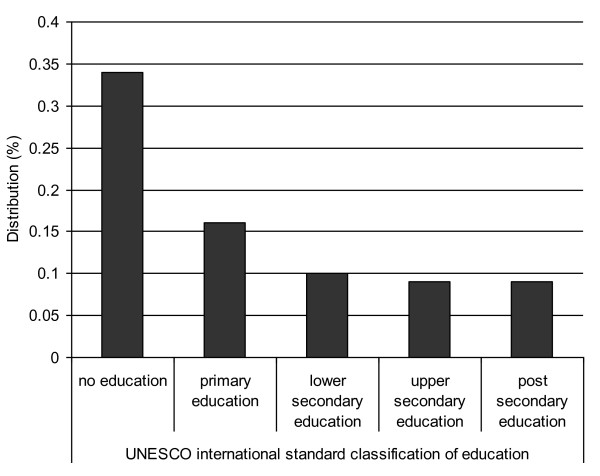
**Distribution of maternal deaths by years of maternal education**.

Figure 1 shows the distribution of maternal deaths by educational category. Findings from the univariate regression analyses in which we modelled associations between maternal mortality and each of the independent variables separately, indicated that the risk of maternal mortality increased with each reduction in educational level (Table 3). There was a statistically significant variation in the risk of maternal mortality among those with fewer than seven compared with those with more than 12 years of education. Women with no education had almost four times the risk (odds ratio 3.92: 95% confidence interval 2.60,5.92) and those with between one and six years of education had almost twice the risk (odds ratio 1.88: 95% confidence interval 1.26,2.79) of maternal mortality compared with women with more than 12 years of education. There was a higher risk of maternal mortality among women giving birth in institutions with less capacity (1.02:1.01,1.03). Institutions with 'less capacity' than others are those with at least one unit less in the institutional capacity index. Higher risk of maternal mortality was also associated with maternal age at birth over 35 (1.79:1.29,2.47), not being married or cohabiting (1.60:1.24,2.07), at least one more previous birth (1.26:1.21,1.31) and at least one unit lower national public expenditure on health care (1.03:1.02,1.04).

**Table 3:** Risk of maternal mortality by individual, institutional and national characteristics

	Unadjusted Odds Ratio (95% CI)
**Characteristics of the women**	
UNESCO International Standard Classification of Education^19^	
Post-secondary/tertiary (>12 years of education)	1.00
Upper secondary (10-12 years of education)	1.05 (0.70,1.57)
Lower secondary (7-9 years of education)	1.14 (0.75,1.73)
Primary education (1-6 years of education)	1.88 (1.26,2.79)
No education	3.92 (2.60,5.92)
	
Maternal age	1.00 (0.98,1.02)
	
Maternal age group	
10-19 years	1.06 (0.76,1.48)
20-25 years	1.00
26-30 years	0.96 (0.74,1.26)
31-35 years	0.86 (0.62,1.21)
36-55 years	1.79 (1.29,2.47)
	
Marital status:	
Married/cohabiting	1.00
Not married/cohabiting	1.60 (1.24,2.07)
	
Higher number of previous births	1.26 (1.21,1.31)
	
**Characteristics of the institutions**	
Lower institutional capacity score	1.02 (1.01,1.03)
	
**Characteristics of the countries**	
Lower national public expenditure on health	1.03 (1.02,1.04)
	
Lower national per capita health expenditure	1.01 (1.00,1.01)

The relationship between lower levels of education and a higher risk of mortality persisted after adjusting for the other potential explanatory factors in the multi-level model, although the difference in mortality risk between no and high levels of education was attenuated in the adjusted model (Table 4). Women with no education had over two and a half times the risk (2.69:1.61,4.50) and those with between one and six years of education had twice the risk (odds ratio 1.98: 95% confidence interval 1.26,3.12) of maternal mortality compared with women with more than 12 years of education, after adjusting for the effects of marital status, maternal age, parity, institutional capacity and levels of state investment in health care. A number of approaches were taken to adjust for the effects of maternal age. There was little variation between the impact of adjusting for quadratic/continuous or categorical indicators of age on the associations between maternal education and mortality. However, a clear indication of the nature of the relationship between maternal mortality and age was best achieved using the categorical measure. Analyses (not shown) suggested that it was the inclusion of parity and, to a lesser extent, national public expenditure on health care which most attenuated the variations in the risk of maternal mortality between those with no and higher levels of maternal education. The Institutional Capacity Index was not associated with maternal mortality in the adjusted model.

**Table 4:** Risk of maternal mortality by individual, institutional and national characteristics: results from the fully adjusted multi-level regression model

Parameter	Odds Ratio (95% CI)
Constant	0.001 (0.00,0.00)
	
**Characteristics of the women**	
UNESCO International Standard Classification of Education^19^	
Post secondary/tertiary (>12 years of education)	1.00
Upper secondary (10-12 years of education)	1.14 (0.74,1.77)
Lower secondary (7-9 years of education)	1.51 (0.96,2.37)
Primary education (1-6 years of education)	1.98 (1.26,3.12)
No education	2.69 (1.61,4.50)
	
Age	
10-19 years	0.89 (0.61,1.30)
20-25 years	1.00
26-30 years	1.00 (0.75,1.32)
31-35 years	1.00 (0.69,1.45)
36-55 years	1.62 (1.06,2.47)
	
Marital status:	
Married/cohabiting	1.00
Not married/cohabiting	1.81 (1.29,2.53)
	
Higher number of previous births	
Scale parameter	1.08 (1.00-1.16)
	
**Characteristics of the institutions**	
Lower institutional capacity score	
Scale parameter	1.01 (0.96-1.06)
	
**Characteristics of the countries**	
Lower national public expenditure on health	
Scale parameter	1.04 (1.01-1.07)
	
Random effect (country)	
Scale parameter σ	1.01 (1.00-1.02)

Being single, separated, divorced or widowed was associated with almost twice the risk of maternal death in the multilevel model compared with those who were married or cohabiting (1.81:1.29,2.53). There was a significantly higher risk of death among those aged over 35 compared with those aged between 20 and 25 years (1.62:1.06,2.47). Higher numbers of previous births (1.08:1.00,1.16) and lower levels of state investment in health care (1.04:1.01,1.07) were also associated with higher risk of maternal death. Additional analyses of the relationships between maternal mortality and number of previous births in the single-level models, using categorical and continuous approaches to its classification (not shown) indicated that there was a significantly higher risk of maternal mortality among those who had previously had four or more births compared with those who had had none. However, this relationship was attenuated in the adjusted model, where there was a significantly higher risk of maternal mortality only among those who had previously had nine or more births, compared with those who had had none.

The odds of maternal mortality were correlated at the country level. The significant random effects co-efficient indicated that there were additional effects on maternal mortality related to country of residence which were not explained by the measures included in the model (1.01:1.00,1.02). The relationships between the random intercept (α) and the random slope (β) were not significant (1.01:0.95,1.08) and there was no heterogeneity across institutions within countries. These findings indicated that variations in maternal mortality between institutions were explained by differences in their Capacity Indices (rather than other differences between them), and that the implications of this capacity for maternal mortality were consistent across countries.

## Discussion

In this international survey of 287,035 women giving birth in health care institutions in 24 countries, women with lower educational levels are more likely to die than women with higher educational levels. Other studies have also reported an association between female education and maternal mortality [27]. However we have demonstrated that, for women who were able to deliver in hospital facilities, the higher mortality of women with lower levels of education cannot be explained by the level of services available at the institution where they gave birth. Maternal age, marital status, parity and level of state investment in health services also had significant independent impacts on maternal mortality. Adjusting for maternal age, marital status, parity and level of state investment in health services does not explain the influence of maternal education on mortality. By delivering in a health care facility, many of these women, particularly those in Africa and Asia, were able to access a level of health resources above that available to many women living in those regions [28]. Yet even for women able to access facilities providing intrapartum care, the impacts of wider social determinants on health (specifically educational level) and mortality persisted.

### Study strengths and limitations

Our study has a number of strengths. These data are in many ways unique. The database includes nearly 300,000 deliveries with information collected in a standardized manner from a large, worldwide network of health care facilities. These data address concerns regarding the limited availability of data on measures of infrastructure and quality in facilities in developing countries [3]. Although the data source is only facility-based and is not representative of the population, the random selection of provinces and facilities within those provinces ensured that there was no selection bias by capacity, proximity or population served. We examined many facilities and regions that had not taken part in a research project of this nature before. Although we were not able to measure the quality of care each woman received during delivery, using a standard measure of capacity across a variety of institutions allowed us to assess whether the services available were able to modify the negative effect of social disadvantage (as measured by education level) on maternal mortality.

A major limitation of this study was the small number of maternal deaths, which may have influenced the reliability of our results. There were three other limitations. First: data availability means that we examined maternal deaths which occurred during the intrapartum period only, and could not include antepartum deaths due to abortion complications or ectopic pregnancy. The potential implications of this restriction is potentially limited as the large majority of maternal deaths take place in this period [22]. This also meant that we are unable to adjust for cause of death, access to antenatal care or medical co-morbidities. Women who died undelivered or who died following discharge from the institution were not included as maternal deaths in the survey. Additional bias may have been introduced if the motivation for women's attendance at health facilities varied by educational level, for example if those with lower education levels attended only following complications while the attendance of other women was more general. Although we adjusted for a number of risk factors for complicated deliveries, we were not able to adjust for morbidities. Finally, our adjustment for national contextual variables is crude and we are unable to take account of potentially important regional and national level influences on maternal mortality, such as the impact of variations in cause of death [22,29-31] or cultural norms, for example those related to childbearing outside marriage [23]. Our results indicated that there were additional influences on maternal mortality, particularly related to country of residence which remain unmeasured and require further investigation.

### Possible explanations for findings

Education may have both a direct and indirect relationship with maternal mortality. Increasing levels of educational attainment are likely to enhance the capacity of women to obtain, process and understand basic health information about the benefits of good prenatal care and the reproductive health services needed to make appropriate health decisions. For example, more educated women may be less likely to accept traditional explanations for life and death and instead take on broad information about birth spacing, the signs of pregnancy complications and the need to improve their nutritional status to reduce the risk of iron deficiency anaemia, all of which are of key importance in the drive to reduce maternal deaths. Furthermore, more educated women are likely to be more confident about asking questions about their health care needs and are more likely to be listened to by health care professionals [12]. The indirect relationship between educational levels and maternal mortality may be through increasing women's self-esteem and thus their empowerment to make health related decisions. Women's improved access to education is also indicative of their more equal position in society [13]. The importance of progress on MDG3 (to promote gender equality and female empowerment, including with regard to education) for the achievement of MDG5 should not be underestimated [32-35]. The relationships between education and status provide more highly educated women with more autonomy to make decisions about the number of children they have, their nutrition during pregnancy and their access to health care [13]. The education of women changes the balance of familial relationships which has profound potential for beneficial effects on maternal mortality. The increased risk of maternal mortality among non-married/cohabiting women is indicative of the ways in which women's social and economic disadvantage combine with attitudes towards childbearing outside marriage to affect women's lives [23].

The increased risk of maternal mortality among women aged over 35 is not specific to countries in Asia, Africa and Latin America [36], and appears to be related to the increased risk of ill health among older women which produce complications during pregnancy and childbirth [37] and the increased likelihood of caesarean delivery at older ages [38]. There is also evidence that the main causes of death vary between women giving birth above and below the age of 35 [39].

## Conclusions

It is accepted that attaining MDG5 depends on widespread improvements in the level and quality of antenatal and obstetric facilities in developing countries. However an exclusive focus on medical and technological approaches to the achievement of this MDG risks overlooking the critical contribution of societal conditions to health [32]. More attention should be given to the wider social determinants of health, including education, and to the factors associated with their interaction with universal health provision, when devising strategies to reduce maternal mortality and to achieve the increasingly elusive MDG for maternal mortality.

## Competing interests

All authors want to declare (1) Financial support for SK for the submitted work from the WHO. Other authors received no financial support for the submitted work from anyone other than their employer. All authors also declare (2) No financial relationships with commercial entities that might have an interest in the submitted work; (3) No spouses, partners, or children with relationships with commercial entities that might have an interest in the submitted work; (4) No non-financial interests that may be relevant to the submitted work.

The authors state their independence from the funders of this work. The funding sources had no role in the study design, analysis or interpretation, the writing of the report or decision to submit the paper for publication. The opinions stated in this paper are those of the authors as individuals, independent from the funding sources. They not necessarily represent the views of the World Health Organization or its member countries.

## Pre-publication history

The pre-publication history for this paper can be accessed here:

http://www.biomedcentral.com/1471-2458/11/606/prepub

## Supplementary Material

Additional file 1 **National maternal mortality and institutional delivery rates**. Information on the maternal mortality and institutional delivery rates for each country included in the analysis, by WHO region. Click here for file
