# Supplementary material for: The relationship between maternal education and mortality among women giving birth in health care institutions: Analysis of the cross sectional WHO Global Survey on Maternal and Perinatal Health
Source: BMC Public Health. 2011 Jul 29;11:606. doi: 10.1186/1471-2458-11-606 (PMC3162526; doi:10.1186/1471-2458-11-606)
Supplement: Additional file 1 — National maternal mortality and institutional delivery rates. Information on the maternal mortality and institutional delivery rates for each country included in the analysis, by WHO region. [file 1471-2458-11-606-S1.DOC]

**Table S1: National maternal mortality and institutional delivery rates^1^**

| Country, by WHO Region | Maternal mortality rate (%, N) | Institutional Delivery Rate  (%, year of reference) |
| --- | --- | --- |
| **Latin America:** |  |  |
| Argentina | 0.05 (10,739) | Information not currently available |
| Brazil | 0.04 (15,150) | 98.4 (2006) |
| Cuba | 0.02 (12,642) | Information not currently available |
| Ecuador | 0.03 (12,414) | 74.1 (2004) |
| Mexico | 0.01 (20,891) | 82.1 (2008) |
| Nicaragua | 0.02 (5,636) | 73.8 (2006-2007) |
| Paraguay | 0.03 (3,522) | 84.6 (2008) |
| Peru | 0.02 (16,041) | 84.4 (2010) |
| **Africa:** |  |  |
| Algeria | 0.04 (15,582) | 95.3 (2006) |
| Angola | 0.58 (6,071) | 42.3 (2008-2009) |
| Democratic Republic of Congo | 0.57 (8,824) | 75.0 (2010) |
| Kenya | 0.17 (19,995) | 42.6 (2008-2009) |
| Niger | 0.10 (8,278) | 17.2 (2006) |
| Nigeria | 0.84 (8,968) | 35.0 (2008) |
| Uganda | 0.22 (13,886) | 41.1 (2006) |
| **Southeast Asia:** |  |  |
| India | 0.17 (24,699) | 46.9 (2007-2008) |
| Nepal | 0.25 (8,493) | 17.7 (2006) |
| Sri Lanka | 0.02 (15,025) | 98.2 (2006-2007) |
| Thailand | 0.03 (9,746) | 99.4 (2009) |
| **Western Pacific:** |  |  |
| Cambodia | 0.05 (5,566) | 53.8 (2010) |
| China | 0.01 (14,544) | 96.3 (2009) |
| Japan | 0.00 (3,301) | 99.8 (2008) |
| Philippines | 0.13 (13,298) | 44.2 (2008) |
| Vietnam | 0.08 (13,309) | 63.8 (2006) |

^1^Data on institutional delivery rates applies to different time periods and employed different data sources in each country. Further details of the data sources utilised are available from the authors.

^2^Southeast Asian and Western Pacific nations were combined for these analyses.
